# Infiltration of Immune Competent Cells into Primary Tumors and Their Surrounding Connective Tissues in Xenograft and Syngeneic Mouse Models

**DOI:** 10.3390/ijms22084213

**Published:** 2021-04-19

**Authors:** Marlon Metzen, Michael Bruns, Wolfgang Deppert, Udo Schumacher

**Affiliations:** 1 Institute of Anatomy and Experimental Morphology, Center for Experimental Medicine, University Cancer Center, University Medical Center Hamburg-Eppendorf, Martinistrasse 52, 20246 Hamburg, Germany; uschumacher@uke.de; 2 Heinrich-Pette-Institute, Leibniz-Institute for Experimental Virology, University Medical Center Hamburg-Eppendorf, Martinistrasse 52, 20246 Hamburg, Germany; mi_bruns@web.de; 3 Heinrich-Pette-Institute, Department of Tumorvirology, University Medical Center Hamburg-Eppendorf, Martinistrasse 52, 20246 Hamburg, Germany; w.deppert@uke.de

**Keywords:** cancer, immune cell infiltration, dendritic cells, macrophages, natural killer cells, T cells, WAP-T mouse model, xenograft mouse model

## Abstract

To fight cancer more efficiently with cell-based immunotherapy, more information about the cells of the immune system and their interaction with cancer cells in vivo is needed. Therefore paraffin wax embedded primary breast cancers from the syngeneic mouse WAP-T model and from xenografted tumors of breast, colon, melanoma, ovarian, neuroblastoma, pancreatic, prostate, and small cell lung cancer were investigated for the infiltration of immunocompetent cells by immunohistochemistry using antibodies against leukocyte markers. The following markers were used: CD45 as a pan-leukocyte marker, BSA-I as a dendritic cell marker, CD11b as an NK cell marker, and CD68 as a marker for macrophages. The labeled immune cells were attributed to the following locations: adjacent adipose tissue, tumor capsule, intra-tumoral septae, and cancer cells directly. In xenograft tumors, the highest score of CD45 and CD11b positive, NK, and dendritic cells were found in the adjacent adipose tissue, followed by lesser infiltration directly located at the cancer cells themselves. The detected numbers of CD45 positive cells differed between the tumor entities: few infiltrating cells in breast cancer, small cell lung cancer, neuroblastoma, a moderate infiltration in colon cancer, melanoma and ovarian cancer, strongest infiltration in prostate and pancreatic cancer. In the syngeneic tumors, the highest score of CD45 and CD11b positive, NK and dendritic cells were observed in the tumor capsule, followed by a lesser infiltration of the cancer tissue. Our findings argue for paying more attention to investigate how immune-competent cells can reach the tumor cells directly.

## 1. Introduction

Cancer remains a considerable health care challenge, with the lifetime risk of getting cancer being 43% for women and 51% for men in Germany (Kobold et al., 2015) [1]. So far, cancer therapy has been based on surgery, radiotherapy, and/or chemotherapy. However, the landscape of cancer therapy is probably changing dramatically with the advent of new immune therapeutic agents. Amongst other reasons, immunotherapy attracted much attention as in some cancers, most of the cells making up the tumor mass have been classified as leukocytes (Allavena et al., 2008, Colotta et al., 2009, Sica et al., 2008) [2,3,4]. Despite this immune cell infiltration, tumor cells are not efficiently attacked as they continue to grow. Unleashing this immune response might therefore be an attractive therapeutic option. Various possible mechanisms in boosting the innate and adaptive immune responses exist. Marquez-Rodas et al., (Marquez-Rodas et al., 2015) [5] and Postow et al., (Postow et al., 2015) [6] described that tumor cells were able to block the attachment of antigen-presenting leukocytes to T lymphocytes, thus stopping their attack capacity by so-called checkpoint mechanisms. These include the blockade of the receptor-ligand-interaction between cytotoxic T lymphocyte antigen-4 (CTLA-4) and CD80/CD86 or between programmed death-1 protein (PD-1) and its ligand (Deppert and Bruns, 2016, Hamid et al., 2011, Krummel and Allison, 1996) [7,8,9].

Common to both adaptive and innate cell-mediated immunity is the direct cytotoxic action to kill cancer cells. The present study focuses on cells of the innate immune system to fill the existing knowledge gap concerning dendritic cells, monocytes/macrophages, and NK cells in xenograft and syngeneic mouse models. The clinical importance of these cell populations was already shown by Horst and Horny (1988) [10] in human ductal breast cancers where monocytes, macrophages, and CD4 positive T cells generally constituted the majority of the infiltrating cells. B lymphocytes and NK cells represented a minority fraction.

The aim of the present study was to collect new data about immune cell infiltration into breast, colon, melanoma, ovarian, neuroblastoma, pancreatic, prostate, and small cell lung cancer in xenograft models of these tumors and to compare them to a syngeneic mouse WAP-T breast cancer model (Bruns et al., 2015, Bruns et al., 2016, Wegwitz et al., 2010) [11,12,13].

## 2. Results

### 2.1. Human Primary Xenograft Tumors

#### 2.1.1. Breast Cancer

In T47D primary tumors, 70% of dendritic cells, no CD45 positive cells, 5% of CD11b positive cells, and no CD68 positive cells were found in the adjacent adipose tissue. In the tumor capsule, 25% of dendritic cells, no CD45 positive cells, no CD11b positive cells, and no CD68 positive cells were detected. In the intra-tumoral septae, 55% of dendritic cells, and none of the other cell populations were detected. In the breast cancer tissue itself, 35% of dendritic cells and none of the other cell populations were found.

In MD-MB-231 primary tumors, 40% of dendritic cells, 40% of CD45 positive cells, 20% of CD11b positive cells, and 15% of CD68 positive cells were found in the adjacent adipose tissue. In the tumor capsule, 20% of dendritic cells, 15% of CD45 positive cells, 30% of CD11b positive cells, and no CD68 positive cells were detected. In the intra-tumoral septae, none of the cell populations were detected. In the breast cancer tissue itself, 30% of dendritic cells, 20% of CD45 positive cells, 15% of CD11b positive cells, and 5% of CD68 positive cells were found.

In DU4475 primary tumors, 70% of dendritic cells, 55% of CD45 positive cells, 5% of CD11b positive cells, and no CD68 positive cells were found in the adjacent adipose tissue. In the tumor capsule, no dendritic cells, 20% of CD45 positive cells, no CD11b positive cells, and no CD68 positive cells were detected. In the intra-tumoral septae, 25% of dendritic cells, 35% of CD45 positive cells, 15% of CD11b positive cells, and 20% of CD68 positive cells were detected. In the breast cancer tissue itself, 65% of dendritic cells, no CD45 positive cells, 20% of CD11b positive cells, and 10% of CD68 positive cells were found.

**Figure 1:**
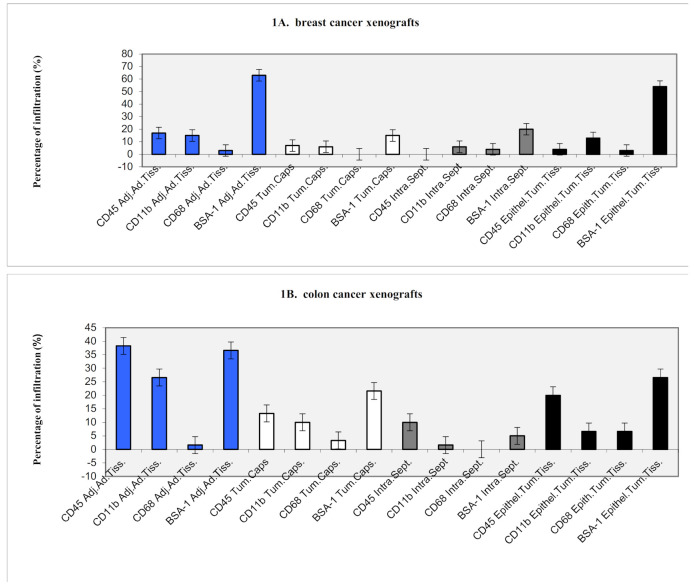

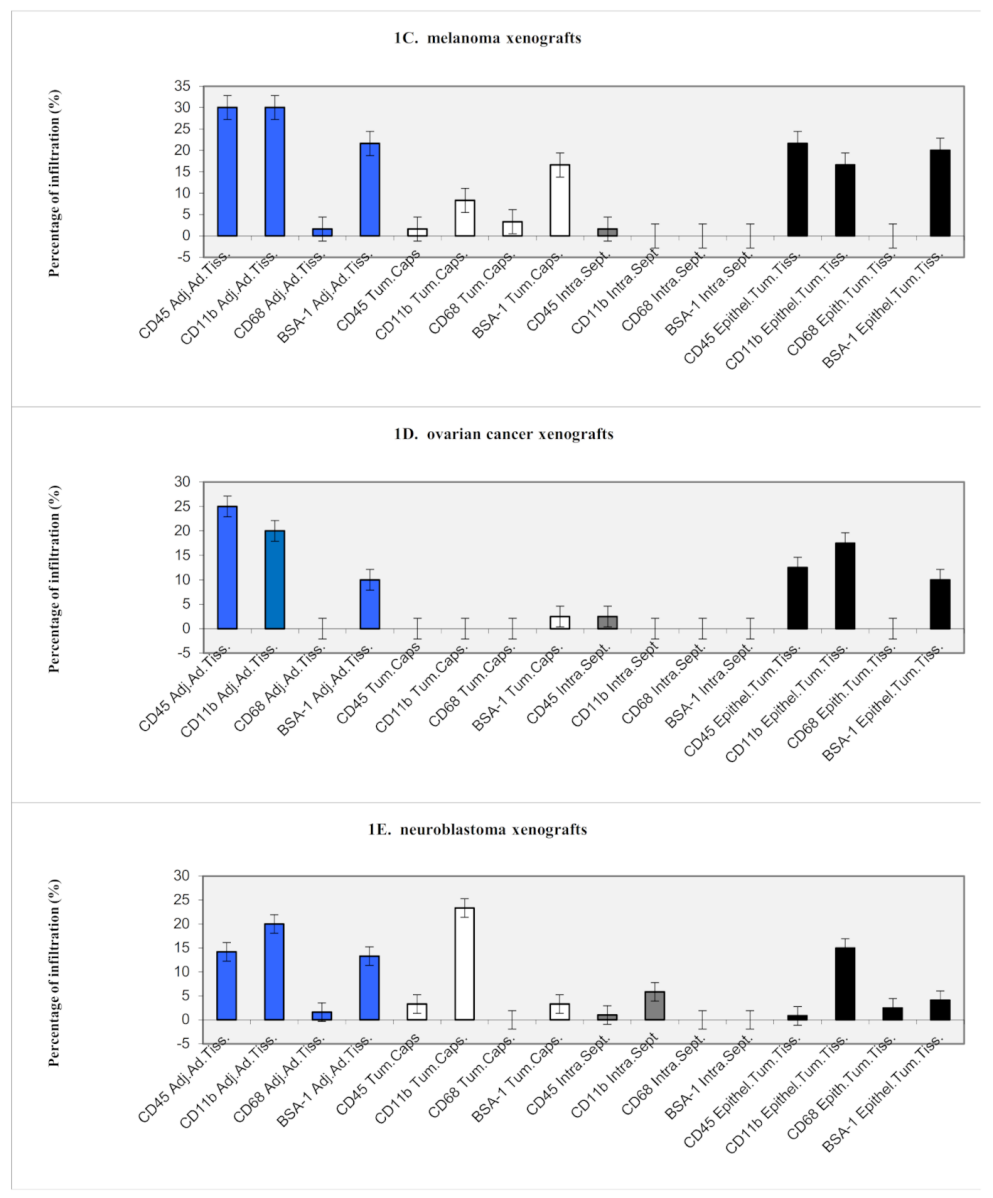

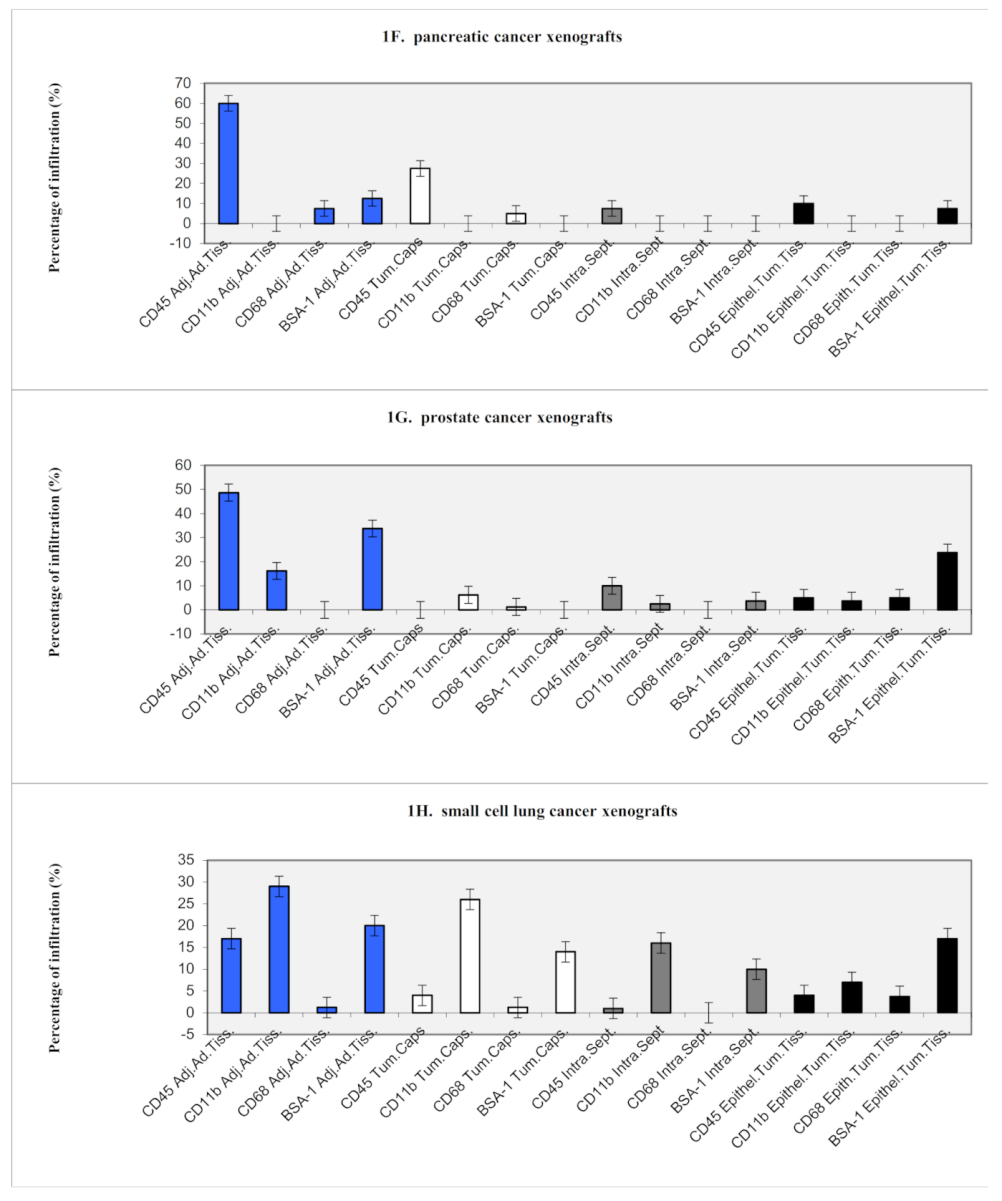
(**A**) breast cancer xenografts, (**B**) colon cancer xenografts, (**C**) melanoma xenografts, (**D**) ovarian cancer xenografts, (**E**) neuroblastoma xenografts, (**F**) pancreatic cancer xenografts, (**G**) prostate cancer xenografts, and (**H**) small cell lung cancer xenografts. Summary of the histological localization of the immune infiltrating cells in the xenograft models. Percentage of cells labeled by selected antigens in scid mice breast cancer, colon cancer, melanoma, ovarian cancer, neuroblastoma, pancreatic cancer, and pfp/rag2 mice prostate cancer and small cell lung cancer, relating to their localization in the adjacent adipose tissue (Adj.Ad.Tiss.), the tumor capsule (Tum.Caps.), the intra-tumoral septae (Intra.Sept.), and epithelial tumor tissue (Epithel. Tum.Tiss.)

**Figure 2:**
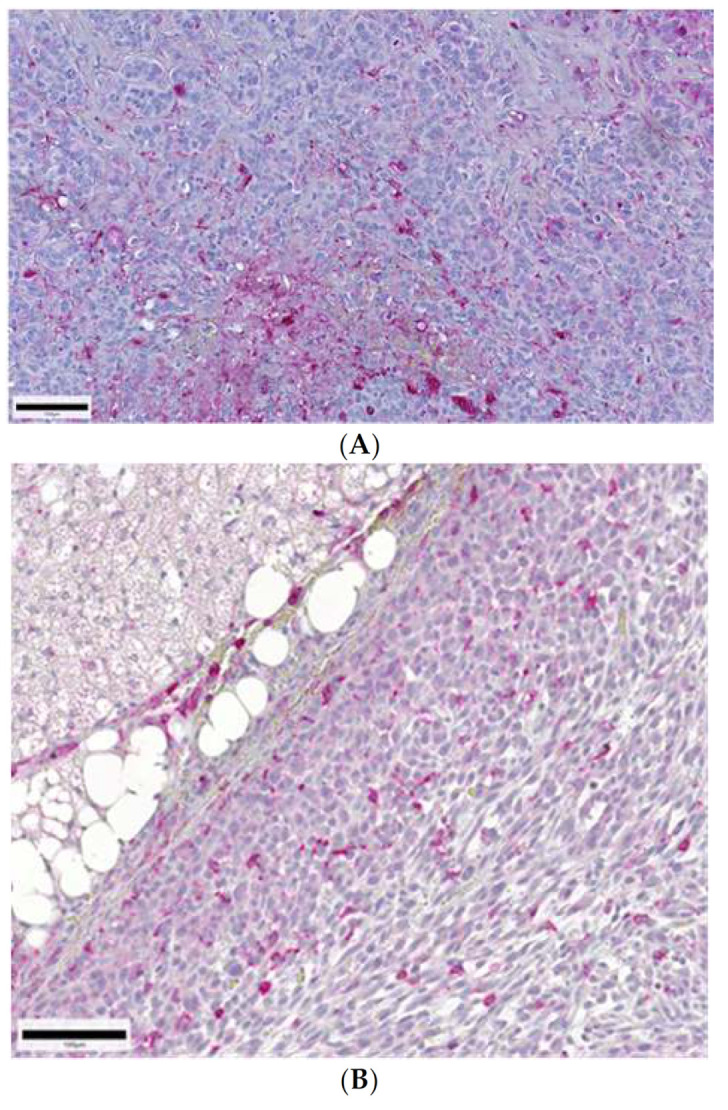

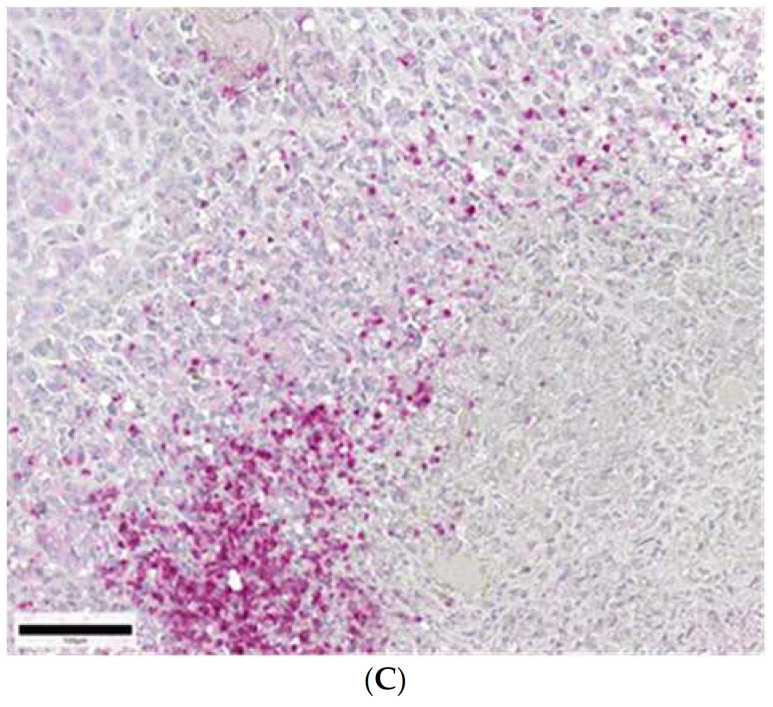
(**A**): Breast cancer (MCF7 105-1A-01). BSA-1 lectin histochemistry in xenograft breast cancer. Tumor-infiltrating dendritic cells were characterized thanks to their high BSA-1 binding capacity. BSA-1 binding cells are considerably detected in all surrounding tissues and moderately in the epithelial tumor tissue itself. (**B**): Neuroblastoma (IMR32 208-6A-06). Tumor-infiltrating natural killer cells (NK cells) in xenograft neuroblastoma were detected thanks to their antibody binding capacity by the rabbit monoclonal anti-CD11b and streptavidin-alkaline phosphate complex with its specific binding capacity for CD11b as an NK cell marker. NK cells were considerably detected in all surrounding tissues but were rare in the epithelial tumor tissue itself. The figure shows an example of a higher infiltration rate. (**C**): Pancreatic cancer (PaCa 5072 256-1A-08). Tumor-infiltrating natural killer cells (NK cells) in xenograft pancreatic carcinoma were detected thanks to their antibody binding capacity by the rabbit monoclonal anti-CD11b and streptavidin-alkaline phosphate complex with its specific binding capacity for CD11b as an NK cell marker. NK cells were considerably detected in all surrounding tissues but were rare in the epithelial tumor tissue itself, as shown in this figure.

In MCF7 primary tumors, 95% of dendritic cells, 5% of CD45 positive cells, 20% of CD11b positive cells, and no CD68 positive cells were found in the adjacent adipose tissue. In the tumor capsule, none of the cell populations were detected. In the intra-tumoral septae, 10% of CD11b positive cells and none of the other cell populations were detected. In the breast cancer tissue itself, 45% of dendritic cells, no CD45 positive cells, 10% of CD11b positive cells, and no CD68 positive cells were found (for a summary of the results, see Figure 1A and Table 1 and Table 2, and for a representation, see Figure 2A).

#### 2.1.2. Colon Cancer

In CaCo primary tumors, 35% of dendritic cells, 10% of CD45 positive cells, 25% of CD11b positive cells, and no CD68 positive cells were found in the adjacent adipose tissue (see Figure 1B). In the tumor capsule, no dendritic cells, 15% of CD45 positive cells, no CD11b positive cells, and no CD68 positive cells were detected. In the intra-tumoral septae, 10% of dendritic cells, 15% of CD45 positive cells, 10% of CD11b positive cells, and no CD68 positive cells were detected. In the colon cancer tissue itself, 10% of dendritic cells, no CD45 positive cells, 5% of CD11b positive cells, and no CD68 positive cells were found.

In SW480 primary tumors, 40% of dendritic cells, no CD45 positive cells, no CD11b positive cells, and no CD68 positive cells were found in the adjacent adipose tissue. In the tumor capsule, 35% of dendritic cells, 5% of CD45 positive cells, 30% of CD11b positive cells, and no CD68 positive cells were detected. In the intra-tumoral septae, 5% of dendritic cells, 5% of CD11b positive cells, and none of the other cell populations were detected. In the colon cancer tissue itself, 50% of dendritic cells, no CD45 positive cells, 10% of CD11b positive cells, and no CD68 positive cells were found.

In LS primary tumors, 5% of dendritic cells, 50% of CD45 positive cells, 5% of CD11b positive cells, and no CD68 positive cells were found in the adjacent adipose tissue. In the tumor capsule, no dendritic cells, 45% of CD45 positive cells, 5% of CD11b positive cells, and no CD68 positive cells were detected. In the intra-tumoral septae, 10% of CD45 positive cells, 10% of CD11b positive cells, and none of the other cell populations were detected. In the colon cancer tissue itself, 5% of dendritic cells, 20% of CD45 positive cells, 30% of CD11b positive cells, and no CD68 positive cells were found.

In HT29 primary tumors, 70% of dendritic cells, 35% of CD45 positive cells, 45% of CD11b positive cells, and 5% of CD68 positive cells were found in the adjacent adipose tissue. In the tumor capsule, 15% of dendritic cells, 55% of CD45 positive cells, 10% of CD11b positive cells, and 5% of CD68 positive cells were detected. In the intra-tumoral septae, 5% of dendritic cells, 25% of CD45 positive cells, 5% of CD11b positive cells, and no CD68 positive cells. In the colon cancer tissue itself, 65% of dendritic cells, 30% of CD45 positive cells, 5% of CD11b positive cells, and 20% of CD68 positive cells were found (for a summary of the results, see Figure 1B and Table 1 and Table 2).

#### 2.1.3. Melanoma

In Fem-X1 primary tumors, 5% of dendritic cells, 25% of CD45 positive cells, 10% of CD11b positive cells, and no CD68 positive cells were found in the adjacent adipose tissue (see Figure 1G). In the tumor capsule, 50% of dendritic cells, 30% of CD45 positive cells, 20% of CD11b positive cells, and 5% of CD68 positive cells were detected. In the intra-tumoral septae, none of the cell populations were detected. In the melanoma tissue itself, 5% of dendritic cells, 20% of CD45 positive cells, 10% of CD11b positive cells, and no CD68 positive cells were found.

In LOXIMVI primary tumors, 25% of dendritic cells, 20% of CD45 positive cells, 75% of CD11b positive cells, and 5% of CD68 positive cells were found in the adjacent adipose tissue. In the tumor capsule, no dendritic cells, 40% of CD45 positive cells, 5% of CD11b positive cells, and 5% of CD68 positive cells were detected. In the intra-tumoral septae, 10% of CD45 positive cells and none of the other cell populations were detected. In the melanoma tissue itself, 50% of dendritic cells, 35% of CD45 positive cells, 40% of CD11b positive cells, and no CD68 positive cells were found.

In MV3 primary tumors, 35% of dendritic cells, 20% of CD45 positive cells, 5% of CD11b positive cells, and no CD68 positive cells were found in the adjacent adipose tissue. In the tumor capsule, no dendritic cells, 15% of CD45 positive cells, no CD11b positive cells, and no CD68 positive cells were detected. In the intra-tumoral septae, 5% of CD45 positive cells and none of the other cell populations were detected. In the melanoma tissue itself, 5% of dendritic cells, 35% of CD45 positive cells, 5% of CD11b positive cells, and no CD68 positive cells were found.

In M-14 primary tumors, 40% of dendritic cells, 5% of CD45 positive cells, 25% of CD11b positive cells, and no CD68 positive cells were found in the adjacent adipose tissue. In the tumor capsule, 25% of dendritic cells, 10% of CD45 positive cells, no CD11b positive cells, and no CD68 positive cells were detected. In the intra-tumoral septae, 20% of dendritic cells, 5% of CD11b positive cells, and none of the other cell populations were detected. In the melanoma tissue itself, 65% of dendritic cells, no CD45 positive cells, 5% of CD11b positive cells, and no CD68 positive cells were found (for a summary of the results, see Figure 1C and Table 1 and Table 2).

#### 2.1.4. Ovarian Cancer

In OC2 primary tumors, 20% of dendritic cells, 25% of CD45 positive cells, 35% of CD11b positive cells, and no CD68 positive cells were found in the adjacent adipose tissue (see Figure 1D). In the tumor capsule, 5% of dendritic cells, 45% of CD45 positive cells, and none of the other cell populations were detected. In the intra-tumoral septae, 5% of CD45 positive cells, 5% of dendritic cells, and none of the other cell populations were detected. In the ovarian cancer tissue itself, 20% of dendritic cells, 5% of CD45 positive cells, 20% of CD11b positive cells, and no CD68 positive cells were found.

In OVCAR3 primary tumors, no dendritic cells, 25% of CD45 positive cells, 5% of CD11b positive cells, and no CD68 positive cells were found in the adjacent adipose tissue. In the tumor capsule, 55% of CD45 positive cells and none of the other cell populations were detected. In the intra-tumoral septae, 5% of CD45 positive cells and none of the other cell populations were detected. In the ovarian cancer tissue itself, no dendritic cells, 30% of CD45 positive cells, 15% of CD11b positive cells, and no CD68 positive cells were found (for a summary of the results, see Figure 1D and Table 1 and Table 2).

#### 2.1.5. Neuroblastoma

In LAN-1 primary tumors, 5% of dendritic cells, 5% of CD45 positive cells, no CD11b positive cells, and 5% of CD68 positive cells were found in the adjacent adipose tissue (see Figure 1E). In the tumor capsule, no dendritic cells, 5% of CD45 positive cells, 25% of CD11b positive cells, and no CD68 positive cells were detected. In the intra-tumoral septae and in the neuroblastoma tissue itself, none of the cell populations were detected.

In IMR31 primary tumors, 35% of dendritic cells, 20% of CD45 positive cells, 55% of CD11b positive cells, and no CD68 positive cells were found in the adjacent adipose tissue. In the tumor capsule, no dendritic cells, 20% of CD45 positive cells, 55% of CD11b positive cells, and no CD68 positive cells were detected. In the intra-tumoral septae, 5% of CD45 positive cells, 10% of CD11b positive cells, and none of the other cell populations were detected. In the neuroblastoma tissue itself, 10% of dendritic cells, no CD45 positive cells, 45% of CD11b positive cells, and no CD68 positive cells were found (for a summary of the results, see Figure 1E and Table 1 and Table 2, and for a representation, see Figure 2B).

#### 2.1.6. Pancreatic Cancer

In PaCa5072 primary tumors, 25% of dendritic cells, 75% of CD45 positive cells, no CD11b positive cells, and 10% of CD68 positive cells were found in the adjacent adipose tissue (see Figure 1F). In the tumor capsule, no dendritic cells, 65% of CD45 positive cells, no CD11b positive cells, and 5% of CD68 positive cells were detected. In the intra-tumoral septae, 5% of CD45 positive cells, 5% of CD68 positive cells, and none of the other cell populations were detected. In the pancreatic cancer tissue itself, no dendritic cells, 10% of CD45 positive cells, no CD11b positive cells, and no CD68 positive cells were found.

In PaCa5061 primary tumors, no dendritic cells, 85% of CD45 positive cells, no CD11b positive cells, and 5% of CD68 positive cells were found in the adjacent adipose tissue. In the tumor capsule, no dendritic cells, 35% of CD45 positive cells, no CD11b positive cells, and 5% of CD68 positive cells were detected. In the intra-tumoral septae, 5% of CD45 positive cells and none of the other cell populations were detected. In the pancreatic cancer tissue itself, 15% of dendritic cells, no CD45 positive cells, no CD11b positive cells, and no CD68 positive cells were found (for a summary of the results, see Figure 1F and Table 1 and Table 2, for a representation, see Figure 2C).

#### 2.1.7. Prostate Cancer

In PC3 primary tumors, 10% of dendritic cells, 55% of CD45 positive cells, 5% of CD11b positive cells, and no CD68 positive cells were found in the adjacent adipose tissue. In the tumor capsule, 80% of CD45 positive cells and none of the other cell populations were detected. In the intra-tumoral septae, 40% of CD45 positive cells and none of the other cell populations were detected. In the prostate cancer tissue itself, none of the cell populations were detected.

In LUCAP primary tumors, 10% of dendritic cells, 30% of CD45 positive cells, 10% of CD11b positive cells, and no CD68 positive cells were found in the adjacent adipose tissue. In the tumor capsule, no dendritic cells, 65% of CD45 positive cells, 10% of CD11b positive cells, and 5% of CD68 positive cells were detected. In the intra-tumoral septae, 5% of CD11b positive cells and none of the other cell populations were detected. In the prostate cancer tissue itself, no dendritic cells, 35% of CD45 positive cells, 15% of CD11b positive cells, and no CD68 positive cells were found.

In DU145 primary tumors, 40% of dendritic cells, 40% of CD45 positive cells, 5% of CD11b positive cells, and no CD68 positive cells were found in the adjacent adipose tissue. In the tumor capsule, no dendritic cells, 70% of CD45 positive cells, 10% of CD11b positive cells, and no CD68 positive cells were detected. In the intra-tumoral septae, 15% of CD45 positive cells and none of the other cell populations were detected. In the prostate cancer tissue itself, 35% of dendritic cells, 5% of CD45 positive cells, no CD11b positive cells, and 15% of CD68 positive cells were found.

In LNCAP primary tumors, 75% of dendritic cells, 60% of CD45 positive cells, 45% of CD11b positive cells, and no CD68 positive cells were found in the adjacent adipose tissue. In the tumor capsule, no dendritic cells, 80% of CD45 positive cells, 5% of CD11b positive cells, and no CD68 positive cells were detected. In the intra-tumoral septae, 35% of CD45 positive cells, 15% of dendritic cells, 5% of CD11b positive cells, and no CD68 positive cells were detected. In the prostate cancer tissue itself, 60% of dendritic cells, 15% of CD45 positive cells, no CD11b positive cells, and 5% of CD68 positive cells were found (for a summary of the results, see Figure 1G and Table 1 and Table 2).

#### 2.1.8. Small Cell Lung Cancer

In SW-2 primary tumors, no dendritic cells, 35% of CD45 positive cells, 50% of CD11b positive cells, and no CD68 positive cells were found in the adjacent adipose tissue. In the tumor capsule, no dendritic cells, 30% of CD45 positive cells, 25% of CD11b positive cells, and no CD68 positive cells were detected. In the intra-tumoral septae, 5% of CD11b positive cells and none of the other cell populations were detected. In the small cell lung cancer tissue itself, none of the cell populations were detected.

In H69AR primary tumors, 25% of dendritic cells, 25% of CD45 positive cells, 55% of CD11b positive cells, and no CD68 positive cells were found in the adjacent adipose tissue. In the tumor capsule, no dendritic cells, 25% of CD45 positive cells, 50% of CD11b positive cells, and no CD68 positive cells were detected. In the intra-tumoral septae, 10% of CD45 positive cells, 10% of dendritic cells, and none of the other cell populations were detected. In the small cell lung cancer tissue itself, 30% of dendritic cells, 15% of CD45 positive cells, no CD11b positive cells, and no CD68 positive cells were found.

In NCI-H69 primary tumors, 35% of dendritic cells, 25% of CD45 positive cells, 40% of CD11b positive cells, and no CD68 positive cells were found in the adjacent adipose tissue. In the tumor capsule, 15% of dendritic cells, 20% of CD45 positive cells, 45% of CD11b positive cells, and no CD68 positive cells were detected. In the intra-tumoral septae, 5% of dendritic cells and 70% of CD11b positive cells and none of the other cell populations were detected. In the small cell lung cancer tissue itself, 40% of dendritic cells, no CD45 positive cells, 10% of CD11b positive cells, and no CD68 positive cells were found (for a summary of the results, see Figure 1H and Table 1; Table 2).

### 2.2. Syngeneic Primary WAP-T1 and WAP-T-NP8 Tumors

#### 2.2.1. Hyperplasia (HP)

**Figure 3:**
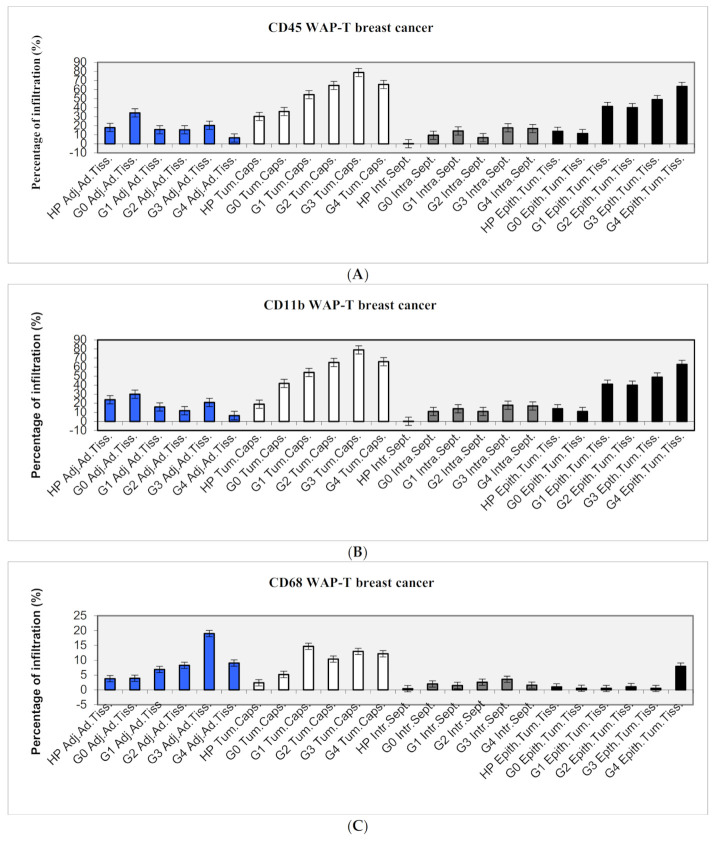

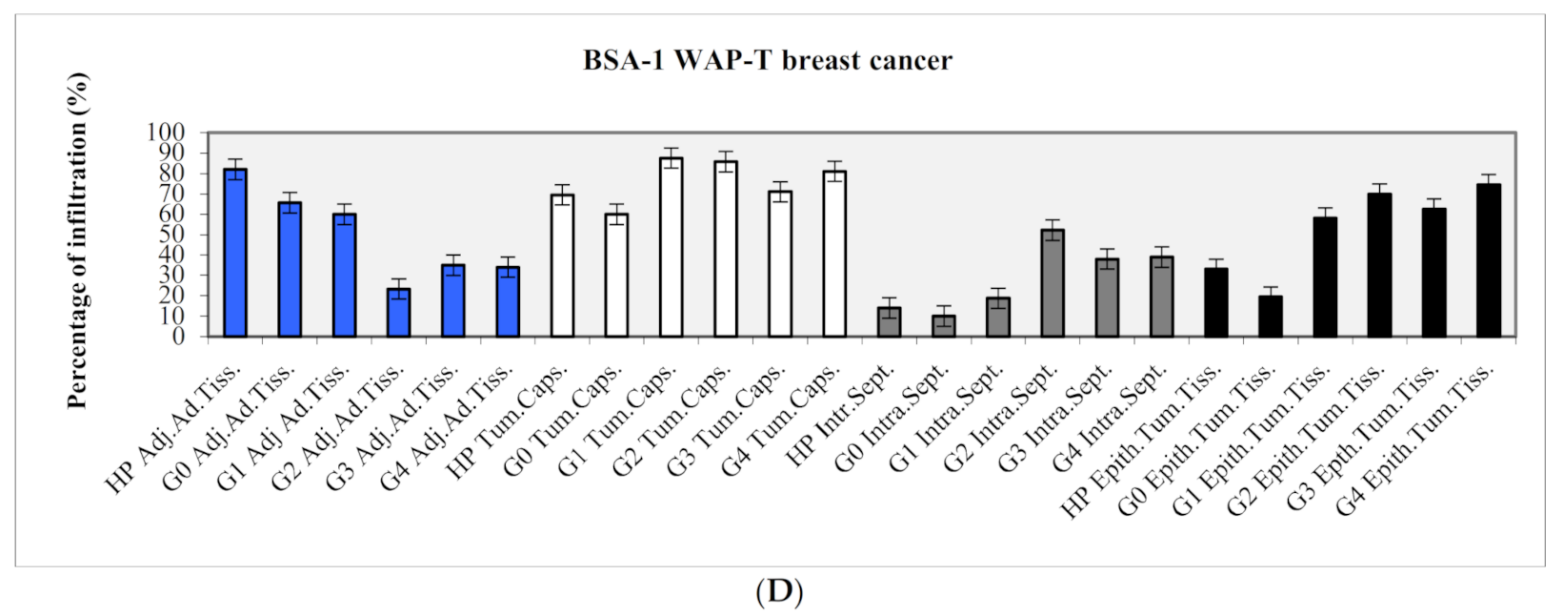
(**A**): CD45 WAP-T breast cancer. Summary of the histological localization of immune infiltrating cells in syngeneic WAP-T mouse breast cancer. (**B**): CD11b WAP-T breast cancer. Summary of the histological localization of immune infiltrating cells in syngeneic WAP-T mouse breast cancer. Percentage of cells labeled by selected antigens in hyperplasia (HP), dysplasia grade 0 (G0), dysplasia grade 1 (G1), dysplasia grade 2 (G2), dysplasia grade 3 (G3), dysplasia grade 4 (G4) relating to their localization in the adjacent adipose tissue (Adj.Ad.Tiss.), the tumor capsule (Tum.Caps.), the intra-tumoral septae (Intra.Sept.) and the epithelial tumor tissue (Epith.Tum.Tiss.). (**C**): CD68 WAP-T breast cancer. Summary of the histological localization of immune infiltrating cells in syngeneic WAP-T mouse breast cancer. Percentage of cells labeled by selected antigens in hyperplasia (HP), dysplasia grade 0 (G0), dysplasia grade 1 (G1), dysplasia grade 2 (G2), dysplasia grade 3 (G3), dysplasia grade 4 (G4) relating to their localization in the adjacent adipose tissue (Adj.Ad.Tiss.), the tumor capsule (Tum.Caps.), the intra-tumoral septae (Intra.Sept.), and the epithelial tumor tissue (Epith.Tum.Tiss.). (**D**): BSA-1 WAP-T breast cancer. Summary of the histological localization of immune infiltrating cells in syngeneic WAP-T mouse breast cancer.

In HP primary tumors, 80% of dendritic cells, 20% of CD45 positive cells, 25% of CD11b positive cells, and 5% of CD68 positive cells were found in the adjacent adipose tissue. In the tumor capsule, 70% of dendritic cells, 30% of CD45 positive cells, 20% of CD11b positive cells, and no CD68 positive cells were detected. In the intra-tumoral septae, 15% of dendritic cells and none of the other cell populations were detected. In the HP tissue itself, 35% of dendritic cells, 15% of CD45 positive cells, 15% of CD11b positive cells, and no CD68 positive cells were found (see Table 2 and Table 3 and Figure 3A–D).

#### 2.2.2. Dysplasia/DCIS (G0)

In G0 primary tumors, 65% of dendritic cells, 35% of CD45 positive cells, 30% of CD11b positive cells, and 5% of CD68 positive cells were found in the adjacent adipose tissue. In the tumor capsule, 60% of dendritic cells, 35% of CD45 positive cells, 40% of CD11b positive cells, and 5% of CD68 positive cells were detected. In the intra-tumoral septae, 10% of dendritic cells, 10% of CD45 positive cells, 10% of CD11b positive cells, and no CD68 positive cells were detected. In the G0 tissue itself, 20% of dendritic cells, 10% of CD45 positive cells, 10% of CD11b positive cells, and no CD68 positive cells were found (see Figure 3A–D and Table 2 and Table 3).

#### 2.2.3. Dysplasia Grade 1 (G1)

In G1 primary tumors, 60% of dendritic cells, 15% of CD45 positive cells, 15% of CD11b positive cells, and 5% of CD68 positive cells were found in the adjacent adipose tissue. In the tumor capsule, 90% of dendritic cells, 55% of CD45 positive cells, 55% of CD11b positive cells, and 15% of CD68 positive cells were detected. In the intra-tumoral septae, 20% of dendritic cells, 15% of CD45 positive cells, 15% of CD11b positive cells, and no CD68 positive cells were detected. In the G1 tissue itself, 60% of dendritic cells, 40% of CD45 positive cells, 40% of CD11b positive cells, and no CD68 positive cells were found (see Figure 3A–D and Table 2 and Table 3).

#### 2.2.4. Dysplasia Grade 2 (G2)

In G2 primary tumors, 25% of dendritic cells, 15% of CD45 positive cells, 10% of CD11b positive cells, and 10% of CD68 positive cells were found in the adjacent adipose tissue. In the tumor capsule, 85% of dendritic cells, 65% of CD45 positive cells, 65% of CD11b positive cells, and 10% of CD68 positive cells were detected. In the intra-tumoral septae, 50% of dendritic cells, 5% of CD45 positive cells, 10% of CD11b positive cells, and no CD68 positive cells were detected. In the G2 tissue itself, 70% of dendritic cells, 40% of CD45 positive cells, 40% of CD11b positive cells, and no CD68 positive cells were found (see Figure 3A–D and Table 2 and Table 3).

#### 2.2.5. Dysplasia Grade 3 (G3)

In G3 primary tumors, 35% of dendritic cells, 20% of CD45 positive cells, 20% of CD11b positive cells, and 20% of CD68 positive cells were found in the adjacent adipose tissue. In the tumor capsule, 70% of dendritic cells, 80% of CD45 positive cells, 80% of CD11b positive cells, and 15% of CD68 positive cells were detected. In the intra-tumoral septae, 40% of dendritic cells, 15% of CD45 positive cells, and none of the other cell populations were detected. In the G3 tissue itself, 60% of dendritic cells, 50% of CD45 positive cells, 50% of CD11b positive cells, and no CD68 positive cells were found (see Figure 3A–D, and Table 2 and Table 3).

#### 2.2.6. Dysplasia Grade 4 (G4)

In G4 primary tumors, 35% of dendritic cells, 5% of CD45 positive cells, 5% of CD11b positive cells, and 10% of CD68 positive cells were found in the adjacent adipose tissue. In the tumor capsule, 80% of dendritic cells, 65% of CD45 positive cells, 65% of CD11b positive cells, and 10% of CD68 positive cells were detected. In the intra-tumoral septae, 40% of dendritic cells, 15% of CD45 positive cells, 20% of CD11b positive cells, and no CD68 positive cells were detected. In the G4 tissue itself, 75% of dendritic cells, 65% of CD45 positive cells, 65% of CD11b positive cells, and 10% of CD68 positive cells were found (see Figure 2A–D and Figure 3E and Table 2; Table 3).

## 3. Discussion

**Figure 4:**
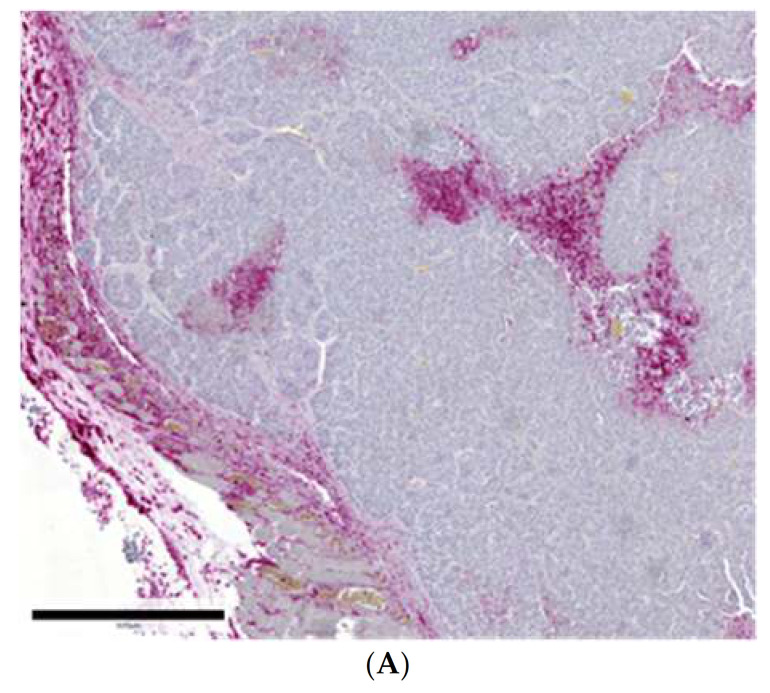

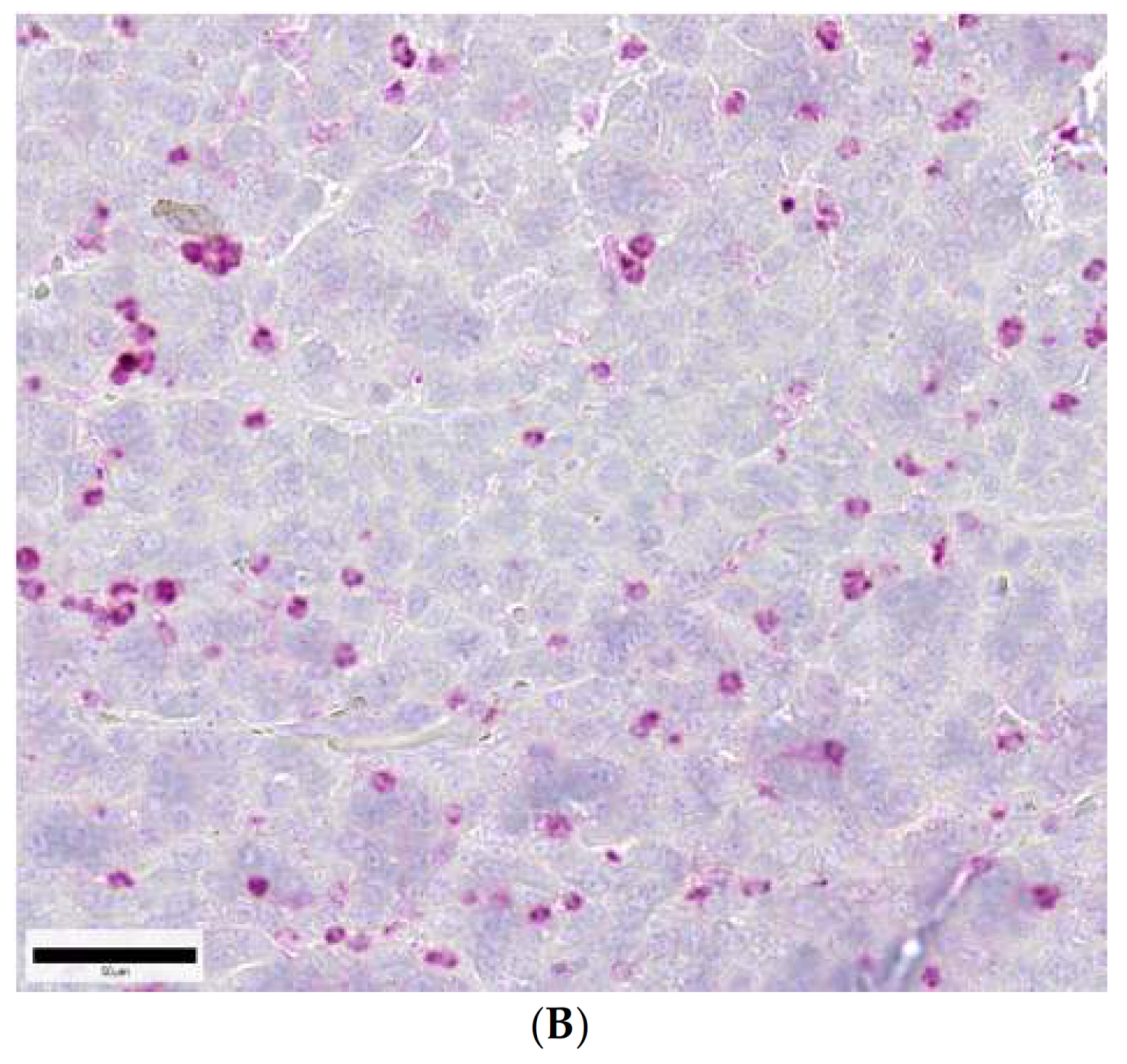
(**A**): Breast cancer dysplasia grade 3 in WAP-T transgenic mice (Syn G3 CD45 135 62.8). Tumor-infiltrating leukocytes were detected thanks to their antibody binding capacity by the antibodies (anti CD45) and the streptavidin-alkaline phosphate complex with its specific binding characteristic for CD45 as a pan-leukocyte marker. Leukocytes are considerably detected in all surrounding tissues and moderate in the epithelial tumor tissue itself, as shown in this figure (**B**): Breast cancer dysplasia grade 4 in WAP-T transgenic mice (Syn G4 CD11b 13333-3). Tumor-infiltrating natural killer cells (NK cells) were detected by the rabbit monoclonal anti-CD11b and the streptavidin-alkaline phosphate complex with its specific binding capacity for CD11b as an NK cell marker. NK cells are considerably detected in all surrounding tissues and moderate in the tumor tissue itself, as shown in this figure.

The present study was performed to examine the quantity and the localization of several different cell populations of the innate and adaptive immune system infiltrating into syngeneic mouse tumors or into human tumors xenografted into immunodeficient mice and to compare them with the results of clinical studies, as immunotherapy of human cancers is currently the most debated aspect of cancer therapy. For studying adenocarcinomas of the breast, the well-established syngeneic WAP-T mouse model was chosen (Bruns et al., 2015, Bruns et al., 2016, Wegwitz et al., 2010, Schulze-Garg et al., 2000) [11,12,13,14], while human tumors were engrafted into severe combined immunodeficient (scid) mice (Custer et al., 1985) [15]. Both WAP-T mice and the strain of scid mice used in our studies were of the BALB/c background, facilitating the direct comparability of the results with regard to the genetic background of the mice. For comparing the results of the distribution of immune-competent cells, the same genetic background is of importance. Therefore any difference in cell populations observed in our experiments can be attributed to the absence/presence of the specific immune system, respectively, only. Our syngeneic WAP-T- breast cancer mouse model reflects the clinical situation well. In a previous study using clinical breast cancer samples, 30% of the tumor-infiltrating immunocompetent cells in ductal carcinoma of the breast were present in the surrounding connective tissue (Horst and Horny, 1988) [10], while 40% of these cells were present in our syngeneic mouse tumors. Similarly, 25% of the infiltrates were detected directly in the epithelial tumor tissues in our WAP-T breast cancers, while it was 19% in the clinical invasive ductal carcinoma cases. Both of these studies showed that immune-competent cells mostly accumulated in the connective tissue septae and in the surrounding adipose tissue while the epithelial tumor mass itself showed a considerably lower degree of infiltration (see Figure 3A–D and Figure 4). These findings stress how well the WAP-T model reflects the situation in humans with regard to immune cell infiltration. The similarity in the global immune cell infiltration between our mouse model and the clinical study is further mirrored by the specific NK cell distribution in human and in mouse tumors. NK cells occurred only in low or very low numbers in the clinical breast cancer study (Horst and Horny, 1988) [10] and similarly were present only in moderate and low numbers or even absent from the analyzed BALB/c WAP-T syngeneic tumors (see Figure 3B). In line with this observation, they were also scarce or absent in scid mouse xenografted tumor tissues implying that the absence of cells of the specific immune system did not have any influence on the presence of NK cells (see Figure 1A). Therefore the scid mouse system could serve as a model system for studying how NK cells infiltrate into solid tumors (see below). In addition, no difference in the presence of NK cells was noted between scid and the perforin knockout pfp/rag2 mouse tumors, indicating that the perforin knockout had no impact on the number of NK cells which were still present albeit in low numbers as in the scid mice. The low number of NK cells in mouse syngeneic tumor tissues was already noted by Van der Weyden (Van der Weyden et al., 2017) [16] who not only noted their low numbers but additionally used a molecular biological approach to boost their numbers in fighting spontaneous lung metastases. Both of these morphological observations corroborate joint experimental and mathematical modeling findings of Brodbeck (Brodbeck et al., 2014) [17], who demonstrated that NK killing of cancer cells mainly takes place in the bloodstream and at the site of the future metastasis rather than in the primary tumor (Brodbeck et al., 2014) [17]. As both Brodbeck (Brodbeck et al., 2014) [17] and Van der Weyden (Van der Weyden et al., 2017) [16] showed that NK cells are crucially involved in anti-tumor responses, our model systems, due to their similarity with the clinical situation, might be ideally suited to investigate how to experimentally increase the influx of NK cells into solid cancer tissues. The influx of immune cells directly adjacent to the cancer cells in solid tumors is obviously limited, with most of the immunocompetent cells being present in the surrounding stroma (‘funnel-effect’). This limited access to the cancer cells directly has already been described in humoral immunity using anti-cancer cell-directed antibodies, which were only distributed about 100μm around blood vessels (Heine et al., 2011, Heine et al., 2012) [18,19]. It has been attributed to the increased interstitial fluid pressure (IFP) within solid tumors (Heine et al., 2012) [19]. As CAR-T cell therapy is quite effective in treating leukemias but is less effective in solid tumors (Wang et al., 2017) [20], it is attractive to speculate that the IFP also, at least in part, hinders the access of immune-competent cells directly to the cancer cells.

In our xenograft scid mouse models, functioning T cells were absent. However, the presence of naïve T lymphocytes in the tumor foci itself has rarely been detected (Kobold et al., 2015) [1] as these adaptive immune cells seem to be halted in their activity and seem to be contained in the surrounding tissues (Kobold et al., 2015, Marquez-Rodas et al., 2015, Postow et al., 2015) [1,5,6]. As T cells are limited in their numbers in cancers, our xenograft models are not too limited in their clinical relevance even if T cells are strongly reduced.

Our model systems should also enable the experimental investigation of the usefulness of the immunoscore (Mlecnik et al., 2016, Galon et al., 2014) [21,22] for the prediction of metastasis formation in our model systems. As the number of spontaneous metastases varies considerably in our xenograft models (see, for example, (Knips et al., 2017, Schwankhaus et al., 2014, Sodeur et al., 2009, Valentiner et al., 2008, Thies et al., 2007, Schumacher and Adam, 1997) [23,24,25,26,27,28]) an immunoscore might help to explain this variation.

## 4. Materials and Methods

### 4.1. Syngeneic WAP-T Mammary Carcinomas

The WAP-T mice were bred and housed under specific pathogen-free conditions at the Heinrich-Pette-Institute, Leibniz-Institute for Experimental Virology Hamburg, Germany, in consensus with official regulations for the care and use of laboratory animals (UKCCCR Guidelines for the Welfare of Animals in Experimental Neoplasia) and approved by Hamburg’s Authority for Health (no.88/06).

The tissues were retrieved from the archives of the Heinrich Pette Institute from a previous experiment (Schreiber et al., 2014) [29].

### 4.2. Xenografted Human Tumors in Scid Mice

The xenografted primary human tumors grown in scid mice were retrieved from the files of the Institute of Anatomy and Experimental Morphology from previous experiments (for a summary, see Table 1). Cell lines: Breast cancer (T47D, MDA-MB231, DU4475 and MCF7 (Schroeder et al., 2010, Schumacher and Adam, 1997, Valentiner et al., 2005) [28,30,31], colon cancer (CaCo, SW480, LS, HT29) (Schumacher and Adam, 1997) [28], melanoma (Fem-X1, LOXIMVI, MV3, M-14) (Thies et al., 2007) [27], ovarian cancer (OC2, OVCAR3) (Oliveira-Ferrer et al., 2014) [32], neuroblastoma (LAN-1, IMR32) (Valentiner et al., 2008) [26], pancreatic cancer (PaCa5072 and PaCa5061) (Gebauer et al., 2013) [33], prostate cancer (PC3, LUCAP, DU145, LNCAP) (Lange et al., 2012) [34] and small cell lung cancer (SW-2, H69AR, NCI-H69) (Sodeur et al., 2009) [25] (see Table 1).

### 4.3. Histology and Immuno- and Lectin Histochemistry

Sections 4 μm thick were attached to adhesion micro slides (Histo Bond; Marienfeld GmbH, Lauda-Königshofen, Germany). The slides were de-paraffinized in xylene and rehydrated through a series of graded ethanols to distilled water. All sections were stained with hematoxylin and eosin according to standard procedures.

Furthermore, the infiltration of lymphocytes, NK cells, and macrophages was analyzed by using monoclonal antibodies against the following antigens: CD45, CD11b, CD68, and BSA-I as a dendritic cell marker. Sections of mouse spleen were always included as positive controls.

### 4.4. Antibody Staining

#### 4.4.1. Pan-Leukocyte Marker CD45

The anti CD45 (BD Pharmingen, # 550539) was diluted 1:25, isotype control antibodies (rat IgG2b 1:400; Biolegend, # 400622) served as a negative control. Deparaffinized sections were heated in a microwave oven (800W) in DAKO retrieval solution (Target Retrieval Solution DAKO by BIOZOL, Germany # S1699). Slides were incubated 1 h at room temperature with the primary antibody, rinsed, and then incubated for 30 min at room temperature with the respective secondary rabbit α rat-biotinylated antibody (Jackson Immuno Research # 312-065-048). CD45 antibody binding sites were detected by incubation of the sections with the streptavidin-alkaline phosphate complex (ABC-AP # AK-5000; Vector Laboratories, Burlingame, CA, USA) for 30 min. For visualizing enzyme reactivity of the alkaline phosphate complex, liquid permanent red (DAKO by BIOZOL, Germany # K0640) was applied to the sections for 15 min under visual control. Then they were washed again under running tap water and finally rinsed in distilled water. After counterstaining with hemalaun, they were finally mounted using a resinous permanent mounting medium (Eukitt, Kindler GmbH, Freiburg, Germany).

#### 4.4.2. NK Cell Marker CD11b

Rabbit monoclonal anti-CD11b (ABCAM, ab # 1259328, Cambridge, UK) was diluted 1:36; rabbit isotype control antibodies served as a negative control (1:400). Sections were de-waxed and pre-treated with citrate buffer (pH 6) and heated for 10 min (121 °C) by using a steamer. After cooling down and washing in TBS-T for 2 × 5 min and 1 × 5 min in TBS, anti-CD11b antibodies (1:200; ABCAM, ab # 133357, Cambridge, UK) were applied, and samples were incubated for 1 h. Thereafter slides were again washed in TBS-T for 2 × 5 min and 1 × 5 min then swine α rabbit (1:200 DAKO # E0353 by BIOZOL, Germany) antibody was added and again incubated for 30 min, washed twice in TBS-T for 5 min and once in TBS for 5 min. Anti-CD11b antibodies were detected by the streptavidin-alkaline phosphate complex as described above.

#### 4.4.3. Macrophage Marker CD68

Anti-CD68M (1:100 end concentration 10 µg/mL, ABCAM ab # 53,444 Cambridge UK) antibodies or isotype controls (rat IgG2a 1:50; eBioscience/Thermo Fisher Scientific, Germany # 14-4321-85) were applied to de-waxed and microwave oven treated slides in DAKO retrieval solution (DAKO by BIOZOL, Germany # S1699) for 30 min.). Prior to antibody application, non-specific binding sites were blocked by incubating the sections in 10% normal rabbit serum 1:10 (DAKO, Germany # XO902) + FcR Blocking Reagent mouse (Miltenyi Biotec, Germany # 130-092-575) for 30 min at room temperature. Slides were incubated with the antibodies or isotype controls, respectively, for 1 h at room temperature. After rinsing in TBS-T and TBS as above, they were incubated for 30 min at room temperature with the respective secondary rabbit- and rat-Biotin antibodies (1:40; Jackson Immuno Research) in TBS. CD68 antibodies were detected by the streptavidin-alkaline phosphate complex, and DAKO liquid permanent red (DAKO) was used for the visualization of the antibody binding site as described above.

#### 4.4.4. Dendritic Cell Marker BSA-I

Dendritic cells were stained by lectin histochemistry with BSA-I (Sigma L2140, Steinheim, Germany), biotinylated lectin from *Bandeiraea simplicifolia* (*Griffonia simplicifolia*) (Thies et al., 2008) [35]. Deparaffinized and rehydrated slides were transferred to 50 mM Tris/HCL with 1 mM MgCl_2_ × 6 H_2_O + 1 mM CaCl_2_ × 2 H_2_O (lectin buffer). Slides were warmed up to 37 °C, and then trypsin (0.1%) was added to the lectin buffer, and the sections were trypsinized for 10 min. Slides were washed 1 × 5 min in tap water, then transferred into lectin buffer 2 × 5 min, then lectin (10 µg/mL) was added and incubated for 1 h. Afterward, slides were washed 3× in TBS for 5 min. Then the slides were incubated with the ABC-Complex (ABC-AP; Vector Laboratories, Burlingame, CA, USA) as above and incubated for 30 min and again washed 3 × 5 min in TBS. For visualizing enzyme reactivity of the alkaline phosphate DAKO liquid, permanent red was used as above. Finally, the slides were counterstained with hemalaun and covered using a resinous permanent mounting medium (Eukitt, Kindler GmbH, Freiburg, Germany). Control sections (negative as well as positive) were incubated in the same way, omitting the lectin in case of the negative control.

The slides were analyzed by a Zeiss Axioplan photomicroscope (Zeiss, Jena, Germany). The histological grading of the WAP-T mouse cancers was classified as described before (Schreiber et al., 2014) [29].

The intensity of staining was scored as follows: (−): no staining; (+): weak; (++): moderate; (+++): strong. The staining pattern of lectin binding was determined for each section in the histological grading. If the staining pattern varied in the sections of one histological grading, the prevailing staining pattern was recorded for the total histological grading.

The percentage of stained immunocompetent cells in each section was estimated at a 5% level and was used to calculate an average value of all slides of each tumor entity. Four different localizations of the immune cells were recorded: (1) adjacent adipose tissue, (2) tumor capsule, (3) intratumoral connective tissue septae, and (4) directly adjacent to the malignant cells.

## 5. Conclusions

In the present study, we immunohistochemically analyzed the distribution and localization of innate and adaptive immune cells both in syngeneic and xenografted tumors. Most of these immunocompetent cells were not, as expected, located in the epithelial tumor tissue itself but were located in the surrounding connective tissues like the adjacent adipose tissue and the tumor capsule. With these results obtained in mice, we corroborate earlier studies using patient material (Horst and Horny, 1988) [10]. In addition to the well-explored checkpoint axes controlled by tumor cells acting on lymphocytes, there should be other reasons for this rare infiltration rate of immune-competent cells into the tumor tissue itself. Perhaps these reasons could be similar to those ones which hamper the access to cancer drugs in solid neoplasms and which include the structure of the tumor blood vessels, the composition of the extracellular matrix, and the increased interstitial fluid pressure (Dewhirst and Secomb, 2017) [36].

## Figures and Tables

**Table 1:** Entities of xenograft tumors with their corresponding cell lines. Scid xenograft tumors (*n* = 464 slides) and pfp/rag2 (*n* = 132 slides).

Entity		Cell Line	(*n*)
Breast cancer	scid	T47D; MDA.MB.231; DU4475; MCF7	106
Colon cancer	scid	CaCo; SW480; LS; HT29	103
Melanoma	scid	Fem-X1; LOXIMVI; MV3; M-14	101
Ovarian cancer	scid	OC2; OVCAR3	51
Neuroblastoma	scid	Lan1; IMR32	52
Pancreatic cancer	scid	PaCa5072; PaCa5061	51
Prostate cancer	pfp/rag2	PC3; LUCAP; DU145; LNCAP	66
Small cell lung cancer	pfp/rag2	SW-2; H69AR; NCI-H69	66

**Table 2:** Legend: color and assignments.

Location	Short Cut	Color
Adjacent adipose tissue	Adj.Ad.Tiss.	blue
Tumor capsule	Tum.Caps.	white
Intratumoral septae	Intra.Sept.	grey
Epithelial tumor tissue	Epithel.Tum.Tiss.	black

**Table 3:** Histological grading of the tissue specimen and sections (*n*) per grading. Histological stages of neoplastic transformation and grading of invasive adenocarcinomas of the breast; number of sections (*n* = 60).

**Hyperplasia (HP)**	**Hyperplasia**	***n* = 10**
Dysplasia/DCIS (G0)	Non-invasive, intraepithelial neoplasia, forms of dysplasia and carcinoma in situ	*n* = 10
Grade 1 (G1)	Well-differentiated invasive adenocarcinoma (low-grade)	*n* = 10
Grade 2 (G2)	Moderately (to poorly) differentiated invasive adenocarcinomas (low-grade)	*n* = 10
Grade 3 (G3)	Poorly differentiated (to undifferentiated) invasive adenocarcinomas (high-grade)	*n* = 10
Grade 4 (G4)	Undifferentiated invasive adenocarcinomas with anaplastic changes (high-grade)	*n* = 10

## Data Availability

The histological slides analyzed during the current study are available from the corresponding author on reasonable request. They are stored for ten years according to the institutional guidelines. The electronic files associated with this project are stored on the UKE (Universitäts Klinikum Hamburg-Eppendorf) servers.
